# Supplementary material for: Integrin α2β1 Represents a Prognostic and Predictive Biomarker in Primary Ovarian Cancer
Source: Biomedicines. 2021 Mar 12;9(3):289. doi: 10.3390/biomedicines9030289 (PMC7999332; doi:10.3390/biomedicines9030289)
Supplement: Supplementary file 1 [file biomedicines-09-00289-s001.pdf]

Integrin $\alpha 2\beta 1$ Represents a Prognostic and Predictive Biomarker in Primary Ovarian Cancer

Katharina Dötzer, Friederike Schlüter, Franz Edler von Koch, Christine E. Brambs, Sabine Anthuber, Sergio Frangini, Bastian Czogalla, Alexander Burges, Jens Werner, Sven Mahner and Barbara Mayer

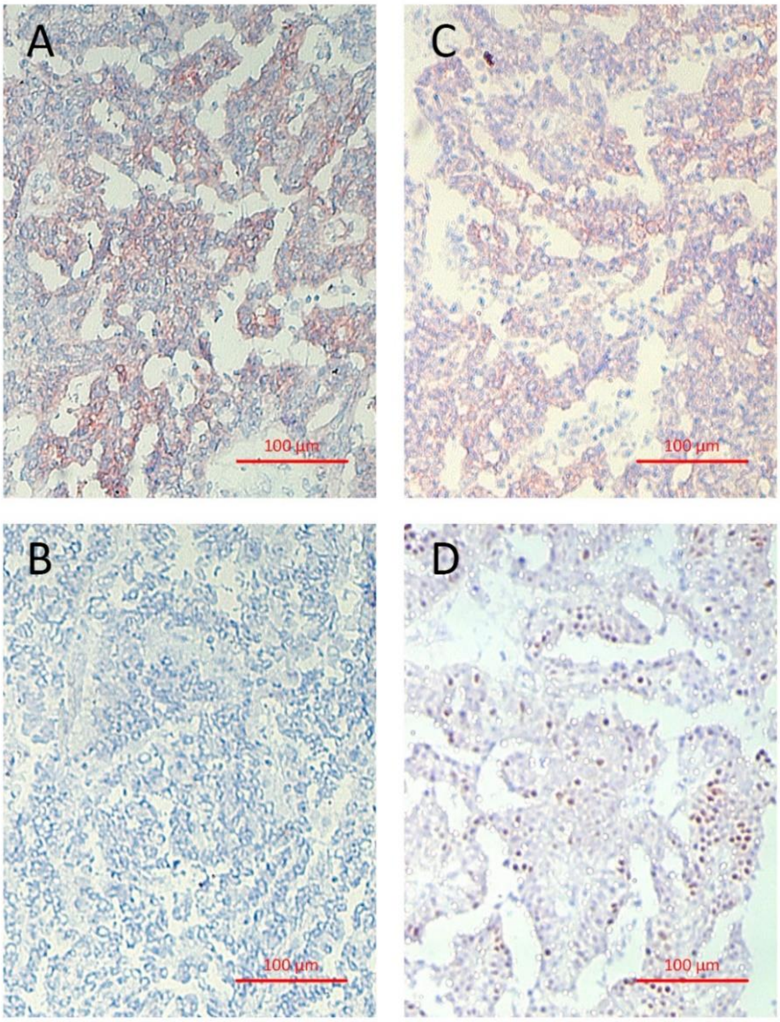

Figure S1. Immunohistochemical Stainings. (**A**) Patient 5197, integrin $\alpha 2\beta 1$, 90% positive cancer cells. (**B**) Patient 5325, integrin $\alpha 2\beta 1$, negative. (**C**) Patient 5197, EGFR, >90% positive cancer cells. (**D**) Patient 5197, ERα, 30% positive cancer cells.
